# Aging aggravates hepatic ischemia-reperfusion injury in mice by impairing mitophagy with the involvement of the EIF2α-parkin pathway

**DOI:** 10.18632/aging.101511

**Published:** 2018-08-08

**Authors:** Yang Li, Dan-yun Ruan, Chang-chang Jia, Jun Zheng, Guo-ying Wang, Hui Zhao, Qing Yang, Wei Liu, Shu-hong Yi, Hua Li, Gen-shu Wang, Yang Yang, Gui-hua Chen, Qi Zhang

**Affiliations:** 1 Department of Liver Surgery and Liver Transplantation, Guangzhou Clinical Research and Translation Center for Liver Disease, The Third Affiliated Hospital of Sun Yat-sen University, Guangzhou 510630, China; 2 Department of Medical Oncology, The Third Affiliated Hospital of Sun Yat-sen University, Guangdong 510630, China; 3 Department of Biotherapy, The Third Affiliated Hospital of Sun Yat-sen University, Guangdong 510630, China; 4 Guangdong Key laboratory of Liver Disease Research, The Third Affiliated Hospital of Sun Yat-sen University, Guangdong 510630, China; * Equal contribution

**Keywords:** aged graft, hepatic ischemia reperfusion injury, mitophagy, graft evaluation, parkin

## Abstract

Hepatic ischemia-reperfusion (I/R) injury fundamentally influences the performance of aged liver grafts. The significance of mitophagy in the age dependence of sensitivity to I/R injury remains poorly understood. Here, we show that aging aggravated hepatic I/R injury with decreased mitophagy in mice. The enhancement of mitophagy resulted in significant protection against hepatic I/R injury. Parkin, an E3 ubiquitin ligase, was found depleted by I/R in aged livers. In oxygen-glucose deprivation reperfusion (OGD-Rep.)-treated L02 cells, parkin silencing impaired mitophagy and aggravated cell damage through a relative large mitochondrial membrane potential transition. The phosphorylation of the endoplasmic reticulum stress response protein EIF2α, which was also reduced in the aged liver, induced parkin expression both in vivo and vitro. Forty-six hepatic biopsy specimens from liver graft were collected 2 hours after complete revascularization, followed by immunohistochemical analyses. Parkin expression was negatively correlated to donor age and the peak level of aspartate aminotransferase within first week after liver transplantation. Our translational study demonstrates that aging aggravated hepatic I/R injury by impairing the age-dependent mitophagy function via an insufficient parkin expression and identifies a new strategy to evaluate the capacity of an aged liver graft in the process of I/R through the parkin expression.

## Introduction

Hepatic ischemia/reperfusion (I/R) is one of the leading causes of liver injury during liver transplantation [1]. Aged donation after cardiac death (DCD) has been proposed as a means of increasing the pool of liver grafts with the aging tendency of the population [2]. The aged liver has a significantly decreased compensatory capacity following I/R. However, the evaluation of the capacity is always a challenge because of an incomplete understanding of the mechanism by which aging increases the sensitivity of the liver to the I/R injury. Among the multifactorial theories, the core pathogenesis is mitochondrial damage and dysfunction [3]. Damaged mitochondria not only compromise the energy metabolism but also produce excessive reactive oxygen species (ROS) and release pro-apoptotic factors, which may ultimately lead to hepatocyte death. Therefore, the maintenance of a cohort of healthy mitochondria is crucial for the homeostasis and viability of hepatocytes in hepatic I/R.

Autophagy is an evolutionarily conserved process that efficiently degrades the dysfunctional organelles in a lysosome-dependent manner [4]. The contribution of autophagy to hepatic I/R injury remains inconclusive. Although several studies have proposed its detrimental roles, most of the evidence supports the idea that autophagy provides cytoprotection against I/R [5]. Mitophagy is an important mechanism of mitochondrial quality control, which selectively removes excessive and defective mitochondria via autophagy. Under stress, mitophagy involves a coordination of autophagy induction and the priming of damaged mitochondria for selective autophagic recognition. Currently, the parkin-dependent pathway is one of the two major pathways of mitochondrial priming for mitophagy. When a subset of mitochondria is damaged and depolarized, PTEN-induced putative kinase 1 (PINK1) accumulates on the mitochondrial outer membrane and recruits parkin from the cytosol and activates its E3 ligase activity via phosphorylation. Upon activation, parkin ubiquitinates various proteins on the outer membrane leading to the recruitment of autophagy receptors to promote the removal of mitochondria via autophagy. In their latest work, Tang demonstrated that PINK1-parkin-mediated mitophagy plays an important role in the mitochondrial quality control, tubular cell survival during I/R induced kidney injury [6]. Moreover, manipulations of the parkin alter the heart’s vulnerability to myocardial infarction [7]. A recent study has shown that tunicamycin limits ischemia-induced brain injury through the induction of parkin-dependent mitophagy [8]. Therefore, we infer that the parkin-dependent mitophagy may protect against hepatic I/R injury.

Autophagy is also a critical mechanism for the aging process. The deletion of autophagic proteins from the liver is associated with early signs of senescence and dysfunction [9]. Defective autophagy has been found in aged hearts and livers [10,11]. Mitochondria are particularly susceptible to age. Abnormalities in the mitochondria are often observed with aging because of the impairment of mitochondrial quality control [12]. The declining mitophagy mediated by parkin is a crucial mechanism for neurodegeneration, arterial stiffness and idiopathic pulmonary fibrosis. These evidences indicate that the defective parkin function during aging may play a significant causative role in age-associated diseases.

Therefore, in the present study, we hypothesize that aging aggravates hepatic I/R injury by impairing age-dependent mitophagy. We further investigate the mechanisms by which mitophagy declines with age in the context of parkin induction.

## RESULTS

### Aging aggravated hepatic I/R injury in C57BL/6 mice

**Figure 1:**
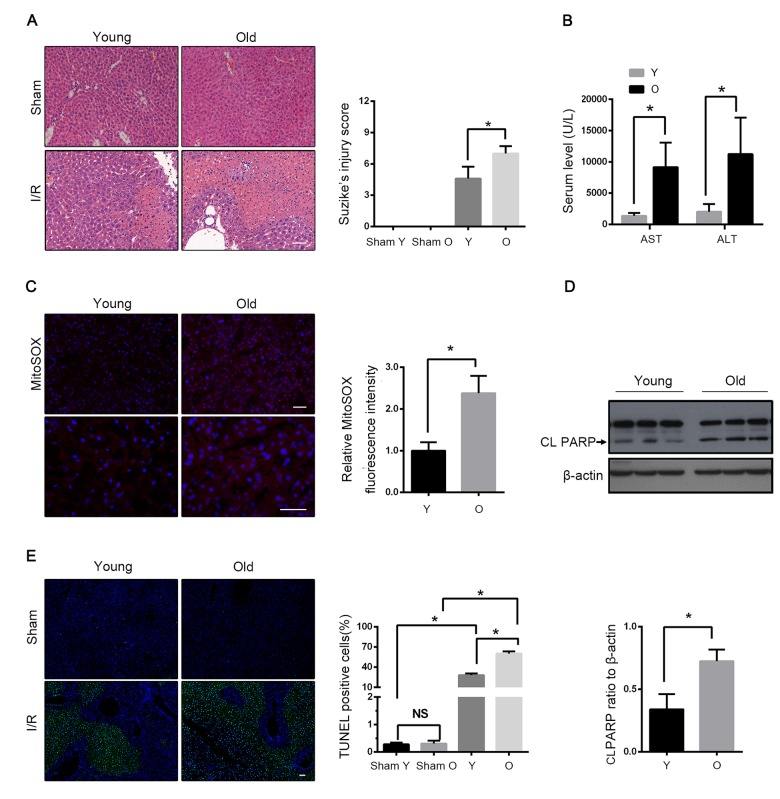
**Aging aggravates hepatic I/R injury in C57BL/6 mice.** Young (Y) and old mice (O) were subjected to middle and left hepatic pedicle occlusion for 1 h before reperfusion (I-R) or sham operation (Sham). Liver and blood were collected 1 h after reperfusion. (**A**) Representative histology of liver by H&E staining and pathological score of liver damage. (**B**) Blood samples were collected for measurements of serum AST and ALT. (**C**) Reactive oxygen species generated by mitochondria was detected by MitoSOX Red staining. (**D**) Whole tissue lysate of liver was collected for immunoblot analysis of cleaved PARP (CL PARP). (**E**) Representative images of TUNEL staining of liver tissues and quantification of TUNEL-positive cells rate in liver tissues. The data are expressed as mean ± SD. Statistical comparisons were performed with t-test. *P < 0.05 vs. the indicated group. Scale bar: 50μm.

Haematoxylin and eosin (H&E) staining revealed that the histological damage of liver after I/R, such as loosening of the hepatocyte cords, was time-dependent in both young and old mice (Fig. S1A). However, the old mice had further marked morphological alterations in liver at 60 min after reperfusion, including the unclear structure of hepatic lobules, disarranged hepatocyte cords, swollen hepatocytes and inflammatory cell infiltration, as compared to young mice (Fig. 1A). Accordingly, the serum levels of alanine aminotransferase (ALT) and aspartate aminotransferase (AST) were prominently increased in old mice as compared to those in young mice (Fig. 1B). To evaluate the level of mitochondrial damage, we examined the mitochondrial ROS by using MitoSOX^tm^ Red, which is a special mitochondrial superoxide indicator. I/R led to a considerably large generation of mitochondrial ROS in mice liver, and the aged liver had relatively large ischemia-induced ROS production (Fig. 1C). In addition, the number of TUNEL-positive cells significantly increased, and apoptosis protein PARP was expressed more in old mice than in young mice (Fig. 1D, E). These data suggested that aging aggravated the hepatic I/R injury in mice.

### Mitophagy declined with age during hepatic I/R

**Figure 2:**
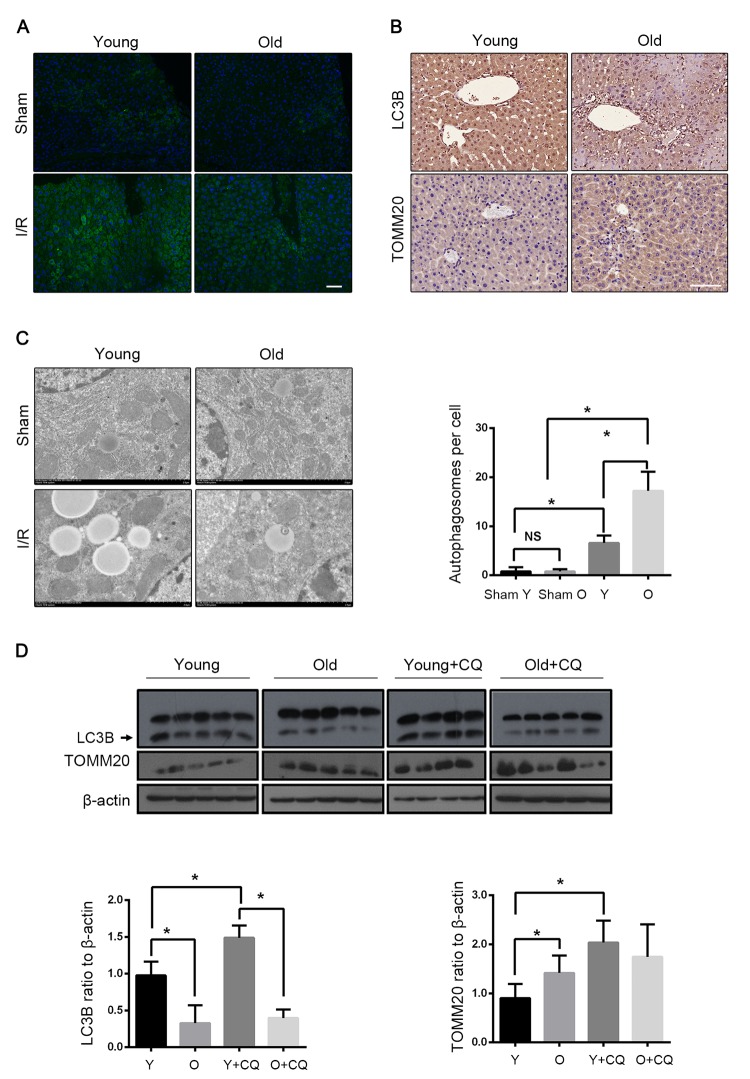
**Mitophagy declines with age during hepatic I/R.** Mice of different age were treated as indicated (Y for young mice, O for old mice, Y+CQ for young mice with chloroquine pretreatment, O+CQ for old mice with chloroquine pretreatment). (**A**) Representative images of LC3B staining of liver tissues by fluorescence microscopy. (**B**) Representative images of LC3B and TOMM20 staining of liver tissues by immunohistochemistry. (**C**) Representative TEM images of autophagosomes in hepatocytes and quantification of autophagosomes in hepatocytes. (**D**) The LC3B and TOMM20 protein levels were determined by western blot analysis from the indicated groups. The data are expressed as mean ± SD. Statistical comparisons were performed with t-test. *P < 0.05 vs. the indicated group, NS no significant difference. Scale bar: 50μm.

To investigate the involvement of autophagy in hepatic I/R, the LC3B expression was assessed. As shown in Fig. 2A and B, LC3B was significantly accumulated in the livers of both young and old mice after I/R, indicating a strong autophagic response to reperfusion. The expression level of LC3B in both the groups gradually increased within 30 min after the reperfusion. However, instead of increasing in young mice, it declined in old mice at 60 min after the reperfusion (Fig. S1B). Meanwhile, an ultrastructural analysis showed that the young mice had a marked autophagosome accumulation as compared to the old mice (Fig. 2C). The immunoblotting of LC3B at 60 min after reperfusion further confirmed that autophagy declined with age in mice liver. Furthermore, the mitochondrial marker TOMM20, which reflects the relative amount of mitochondria, was found to have reduced more in the young group than in the old group, correspondingly (Fig. 2B, D). To confirm that the mitochondria loss was autophagic, the lysosome inhibitor chloroquine was used, which significantly reversed the reduction of TOMM20 in the young mice. In contrast, this was not observed in the old mice, which implies that mitophagy is defective in the old mice after hepatic I/R. Reperfusion following ischemia provoked an increase in the LC3B expression in the old mice, which was not altered in the presence of chloroquine, suggesting a markedly impaired autophagic flux in the old mice (Fig. 2D). To further investigate the effects of aging on hepatocellular mitophagy, we examined LC3B and TOMM20 under the non-ischemic condition. The immunoblotting of LC3B and TOMM20 showed a similar basal level between different age groups (Fig. S1D). These findings suggested that mitophagy declined with age in mice liver following hepatic I/R.

### Mitophagy protected liver from I/R injury

**Figure 3:**
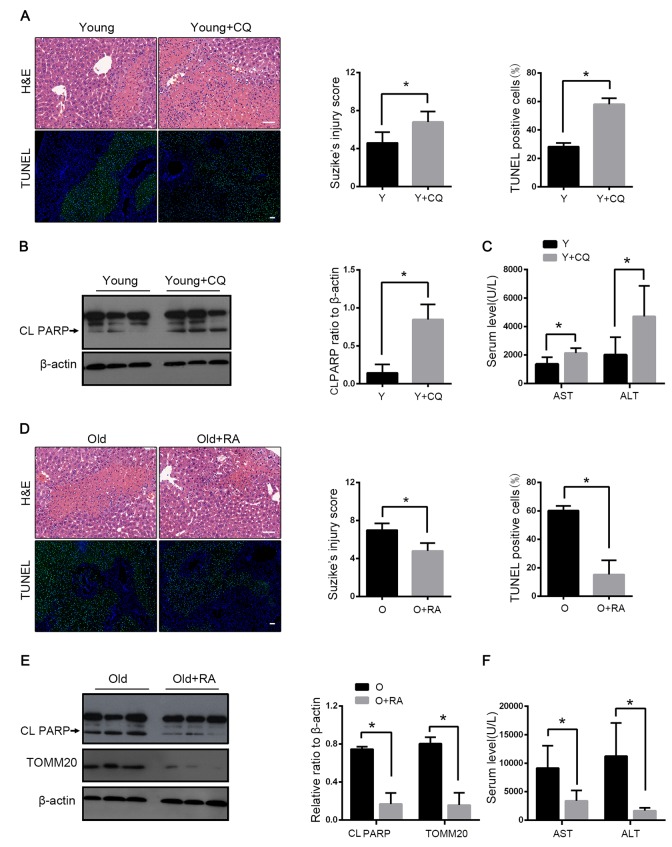
**Mitophagy protects liver from I/R injury.** Young mice with CQ pretreatment (Y+CQ) or without (Y) were treated as indicated. (**A**) Representative histology of liver by H&E staining (top panel) and pathological score of liver damage from indicated groups; TUNEL staining (bottom panel) and quantification of TUNEL-positive cells rate from indicated groups. (**B**) The cleaved PARP (CL PARP) protein levels were determined by western blot analysis from indicated groups. (**C**) Measurements of serum AST and ALT from indicated groups. Old mice with Rapamycin pretreatment (O+RA) or without (O) were treated as indicated. (**D**) Representative histology of liver by H&E staining (top panel) and pathological score of liver damage from indicated groups; TUNEL staining (bottom panel) and quantification of TUNEL-positive cells rate from indicated groups. (**E**) The cleaved PARP (CL PARP) and TOMM20 protein levels were determined by western blot analysis from indicated groups. (**F**) Measurements of serum AST and ALT from indicated groups. The data are expressed as mean ± SD. Statistical comparisons were performed with t-test. *P < 0.05 vs. the indicated group. Scale bar: 50μm.

We detected the effect of autophagy on mice hepatic I/R by using the autophagy inhibitor chloroquine (CQ) and the autophagy activator rapamycin. With the CQ pretreatment, the mitophagy flux was blocked as reflected by the increased LC3B and the decreased TOMM20 levels (Fig. 2D). The histopathological investigation of the liver sections showed worse results in the case of CQ pretreatment with more apoptosis than in the case of the control group. Correspondingly, the immunoblot analysis suggested that the CQ pretreatment increased the expression of the apoptotic protein PARP and more apoptosis was observed (Fig. 3A, B). The serum levels of ALT and AST mirrored the results of the pathological founding (Fig. 3C). These data suggested that the inhibition of mitophagy aggravated hepatic I/R injury. To verify the observation, we tested the effects of the autophagy activator rapamycin in the old mice. Consistently, the serum enzyme, H&E and TUNEL staining showed that rapamycin attenuated the I/R injury in the old mice (Fig. 3D, F). As shown in Fig. 3E, rapamycin significantly down-regulated PARP with less TOMM20, which indicated that rapamycin could alleviate I/R-induced apoptotic cell death in old mice liver by enhancing mitophagy. Taken together, these results further confirmed that mitophagy played a protective role in hepatic I/R injury.

### Parkin and Atg5 reduced in the livers of old C57BL/6 mice during hepatic I/R

**Figure 4:**
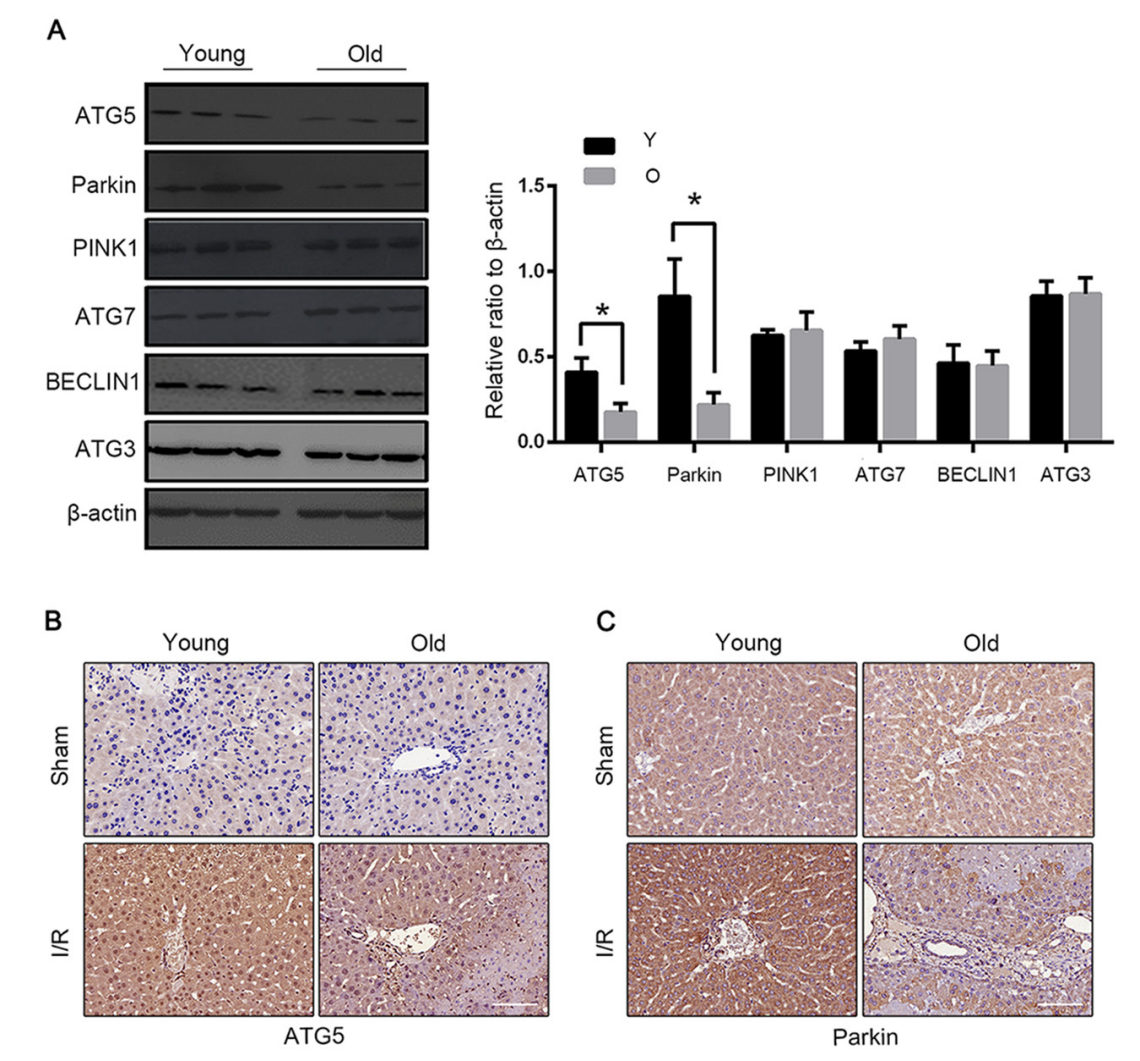
**Parkin and Atg5 reduced in livers of old C57BL/6 mice during hepatic I/R.** (**A**) The Atg5, Parkin, PINK1, Atg7, BECLIN1 and Atg3 protein levels were determined by western blot analysis from the liver tissue of young (Y) and old (O) mice. Representative images of Atg5 (**B**) and Parkin (**C**) staining of liver tissues by immunohistochemistry. The data are expressed as mean ± SD. Statistical comparisons were performed with t-test. *P < 0.05 vs. the indicated group. Scale bar: 50μm.

We next explored the cellular mechanisms underlying the defective mitophagy in the reperfused liver of old mice. Parkin has been demonstrated to play a crucial role in mitophagy induction by ubiquitinating mitochondrial proteins. Atg5 is essential in normal autophagy progression through the modification of LC3. Parkin and Atg5 expressions substantially declined in old mice after I/R. The expressions of Atg7, Atg3, Beclin1 and PINK1 were similar between the young and the old groups (Fig. 4A). Consistent with the expression of LC3B, the expressions of parkin and Atg5 also centred in the vascular area (Fig. 4B, C).

### Parkin protected L02 cells from OGD-Rep-induced injury

**Figure 5:**
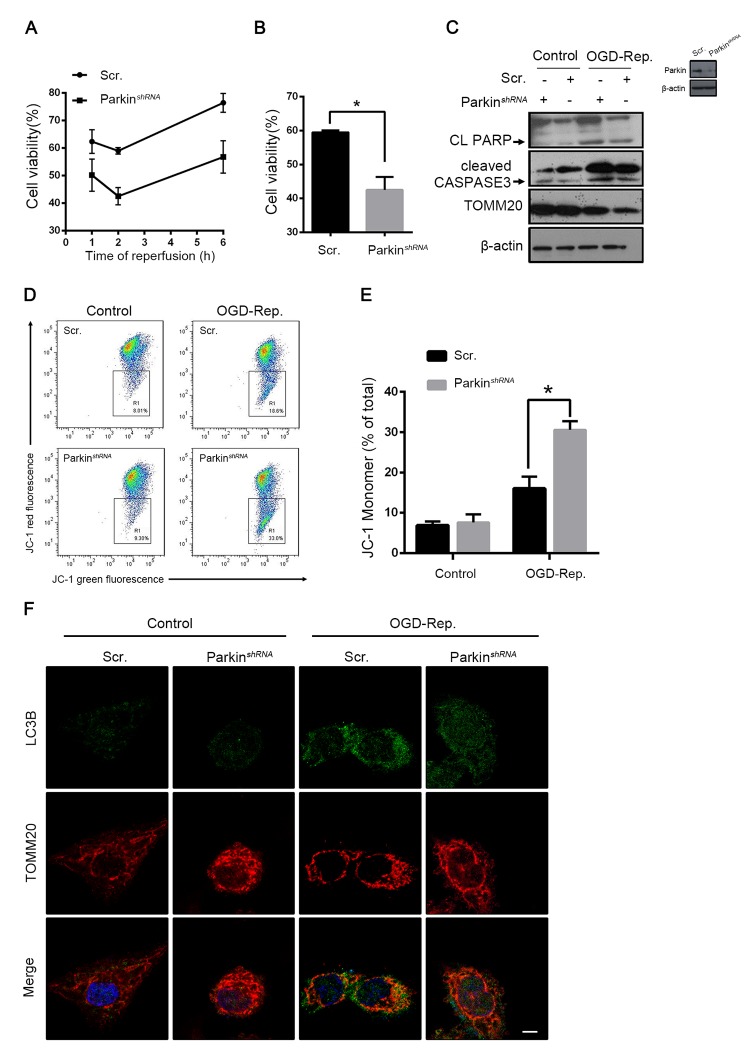
**Parkin protected L02 cells from OGD-Rep. induced injury.** L02 cells were transfected with Parkin shRNA (Parkin *^shRNA^*) or control shRNA (Scr.). Transfected L02 cells were subjected to OGD for 24 h, followed by recovery in normal cell culture medium and oxygen for reperfusion. (**A**) Cell viability was detected by CCK8 at 1, 2 and 6 h after reperfusion. (**B**) Quantification of cells viability at 2 h after reperfusion from indicated cells. (**C**) At 2 h after reperfusion, the whole cells lysate was collected. Cleaved PARP (CL PARP), cleaved CASPASE-3 and TOMM20 protein levels were determined by western blot analysis. (**D**) Measurement of mitochondrial membrane potential by JC-1 flow cytometry at 2 h after reperfusion. (**E**) Quantification of mitochondrial membrane potential loss. (**F**) LC3B (green) and the mitochondrial marker TOMM20 (red) were stained by immunofluorescence and the images were taken by confocal microscopy after 2 h of reperfusion. The data are expressed as mean ± SD. Statistical comparisons were performed with t-test. *P < 0.05 vs. the indicated group. Scale bar: 10μm.

To explore the involvement of parkin in hepatic I/R injury, the L02 cells were subjected to an oxygen-glucose deprivation (OGD) treatment for 24 h. The reperfusion was performed by refreshing the cells with a normal medium and oxygen. As revealed by the results shown in Fig. 5A and B, the interfered parkin expression in the L02 cells decreased the cell vulnerability to the OGD-Rep insult. Similar to the results *in vivo*, the cell viability was significantly reduced in the parkin-silenced cells. Cleaved caspase 3 and PARP were overexpressed accordingly, which reflected that parkin protected against OGD-Red-induced apoptotic cell death (Fig. 5C). To determine the involvement of mitochondria-dependent apoptosis, the mitochondrial membrane potential was examined. The results showed that parkin partly reversed the membrane potential loss revealed by the JC-1 flow cytometry (Fig. 5D, E). To address the function of parkin in OGD-Rep, the overlap between mitochondria and LC3B was quantified. We found that parkin silencing significantly impaired the mitochondria clearance, which was also reflected by the relative TOMM20 level (Fig. 5C, F). Therefore, these results indicated that parkin protected the L02 cells from the OGD-Rep injury through mitophagy.

### Phosphorylation of EIF2α induced mitophagy in L02 cells after OGD-Rep. with increased parkin expression

**Figure 6:**
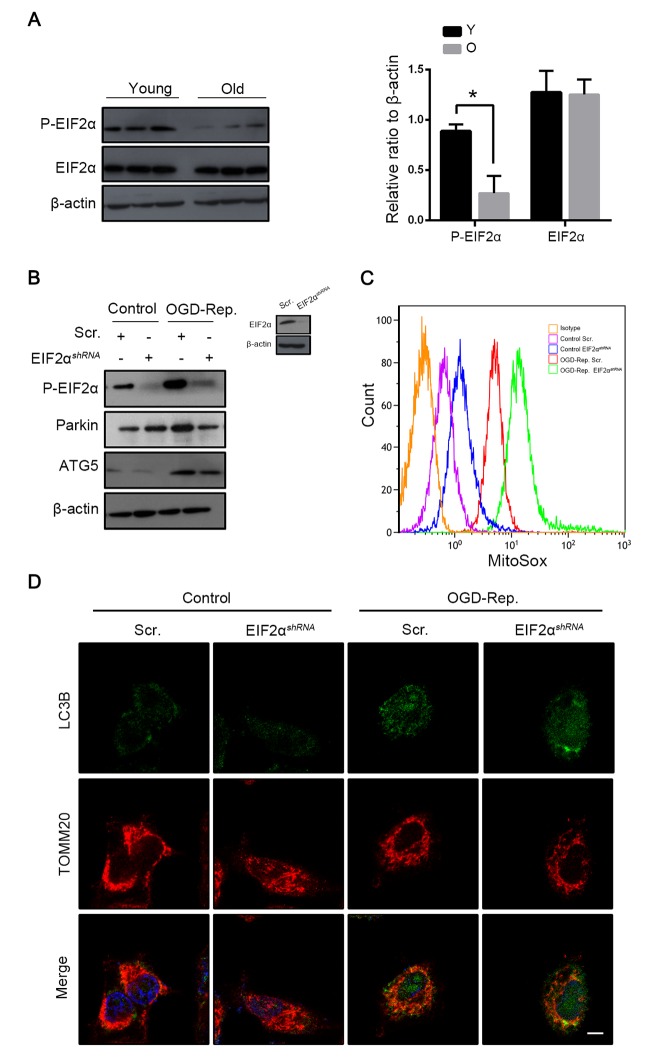
**Phosphorylation of EIF2α induced mitophagy in L02 cells after OGD-Rep. with increased parkin expression.** Mice of different age were treated as indicated (Y for young mice, O for old mice). (**A**) The EIF2α and phosphorylated EIF2α protein levels were determined by western blot analysis from the indicated groups. L02 cells were transfected with EIF2α shRNA (EIF2α*^shRNA^*) or control shRNA (Scr.). Transfected L02 cells were subjected to OGD for 24 h, followed by recovery in normal cell culture medium and oxygen for 2 h. (**B**) The whole cells lysate was collected and phosphorylated EIF2α, Parkin and Atg5 protein levels were determined by western blot analysis. (**C**) Mitochondria reactive oxygen species generation by was detected by MitoSOX^red^ flow cytometry. (**D**) LC3B (green) and the mitochondrial marker TOMM20 (red) were stained by immunofluorescence. and the images were taken by confocal microscopy after 2 h of reperfusion. The data are expressed as mean ± SD. Statistical comparisons were performed with t-test. *P < 0.05 vs. the indicated group. Scale bar: 10μm.

The PERK-EIF2α pathway of ER stress response has been demonstrated to play a crucial role in mitophagy induction in mammalian cells in the context of I-R [8]. To further explore the mechanisms by which parkin was downregulated in old mice, the PERK-EIF2α signaling pathway was investigated. *In vivo*, as with parkin, the phosphorylation of EIF2α was more highly expressed in young mice than in aged mice after hepatic I/R (Fig. 6A). To further confirm that phosphorylated EIF2α regulates the parkin expression, we interfered the EIF2α expression in the L02 cells and found that parkin was downregulated simultaneously after the OGD-Rep insult and more ROS was generated by the mitochondria (Fig. 6B, C). In addition, immunofluorescence revealed that the recruitment of LC3B to the mitochondria in the OGD-Rep-treated L02 cells was reduced after the knockdown of EIF2α (Fig. 6D). Overall, these data indicated that the phosphorylation of EIF2α protects the cells from the OGD-Rep insult through mitophagy by the induction of parkin.

### Salubrinal alleviated HIRI through phosphorylation of EIF2α

**Figure 7:**
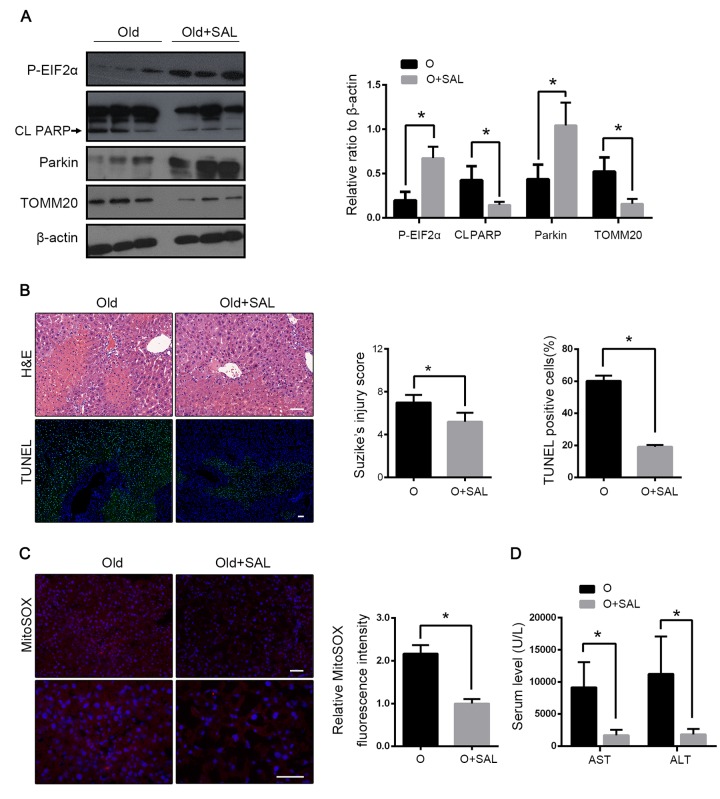
**Salubrinal alleviated HIRI through phosphorylation of EIF2α.** Old mice with Salubrinal pretreatment (O+SAL) or without (O) were treated as indicated. (**A**) The phosphorylated EIF2α, cleaved PARP (CL PARP), Parkin and TOMM20 protein levels were determined by western blot analysis from indicated mice. (**B**) Representative histology of liver by H&E staining (top panel) and TUNEL staining (bottom panel) from indicated groups. (**C**) Representative reactive oxygen species generation by MitoSOX Red staining and quantification of MitoSOX red fluorescence intensity. (**D**) Measurements of serum AST and ALT from indicated groups. The data are expressed as mean ± SD. Statistical comparisons were performed with t-test. *P < 0.05 vs. the indicated group. Scale bar: 50μm.

To clarify the interaction between EIF2α and parkin in hepatic I/R, salubrinal was administered in old mice before hepatic I/R. Salubrinal is an inhibitor of the protein phosphatase PP1 and maintains the high phosphorylation of EIF2α. Salubrinal-pretreated mice showed a higher expression of phosphorylated EIF2α, reversion of parkin reduction and enhancement of mitophagy as revealed by the expression of TOMM20 after reperfusion (Fig. 7A). With the increased mitochondrial clearance, the production of mitochondrial ROS was reduced, which led to less hepatocyte apoptosis (Fig. 7B, C). In mice with hepatic I/R, the histological damage and the transaminase level were also reduced with the salubrinal pretreatment (Fig. 7D). Taken together, these data suggested that the reduced EIF2α phosphorylation may be involved in the parkin downregulation in the old mice following hepatic I/R and subsequently, aggravate the hepatocyte damage by impairing the mitophagy induction.

### Parkin predicted allograft I/R injury after liver transplantation

**Figure 8:**
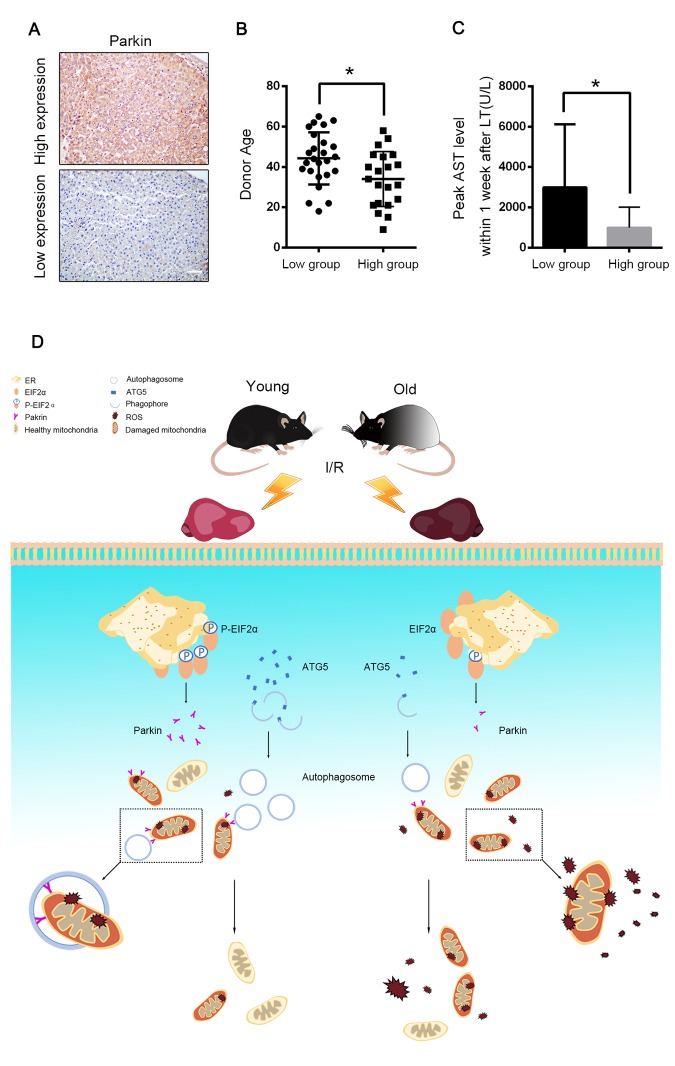
**Parkin predicted allograft I/R injury after liver transplantation.** 46 graft biopsies were performed 2 hours after complete revascularization in 46 patients undergoing DCD liver transplantation. The data of donor age and peak AST within 7 days after transplantation were collected. (**A**) Representative images of Parkin expression in liver graft by immunohistochemistry, 21 patients were in Parkin high-expression group and 25 patients were in Parkin-low expression group. Scale bar: 50μm. (**B**) The donor age of low-expression group was significantly older than high-expression group (44.2±2.6 vs. 34.0±3.0, p<0.05.) (**C**) The peak AST within 7 days after transplantation of low-expression group were significantly higher than high-expression group (2991±624.4 U/L vs. 993.6±221.8 U/L, p<0.01). (**D**) Aging aggravated hepatic I/R injury by impairing age-dependent mitophagy function via insufficient Parkin and Atg5 expression**.** Atg5 decreases in old reperfused liver leading to less formation of autophagosomes. Reperfusion of old ischemic mice liver decreases phosphorylation of EIF2α, which in turn inhibits Parkin expression. Reduced parkin expression and autophagosomes formation subsequently impairs mitophagy and promotes onset of the MPT and cell death. Atg5 and Parkin deficiency is responsible for age-dependent mitophagy impairment.

A graft biopsy was performed 2 h after the complete revascularisation in the 46 patients who underwent DCD liver transplantation. All the patients were classified into two groups depending on their parkin expression in the allograft. 21 patients were defined as the high-expression group and 25 patients as the low-expression group (Fig. 8A). The donor age of the low-expression group was significantly higher than that of the high-expression group (44.2 ± 2.6 years *vs.* 34.0 ± 3.0 years, *p* < 0.05, Fig. 8B). The peak AST within 7 days after the transplantation was also negatively correlated to the allograft parkin expression (2991 ± 624.4 U/L *vs.* 993.6 ± 221.8 U/L, for the low- and the high-expression groups, respectively, *p* < 0.01, Fig. 8C). These data further indicated the relationship among parkin expression, donor age and I/R injury in the cases of liver transplantation.

## DISCUSSION

Hepatic I/R injury profoundly influences the burden of liver diseases. As life expectancy continues to increase, we are facing a drastically increased risk with elderly patients as potential donors for liver transplantation because of the vulnerability to pathological stresses in aged liver grafts [13]. In the present investigation, we demonstrated that defective mitophagy, as a consequence of parkin and Atg5 reduction, is a causal mechanism for the age-dependent hepatic I/R injury (Fig. 8D) and the induction of parkin expression by maintaining the phosphorylation of EIF2α has a therapeutic potential for ameliorating the age-mediated hepatic I/R injury.

Ischemia-induced energy depletion rapidly disrupts the mitochondria and ultimately results in cell death [14]. Some of the damaged mitochondria were normally sequestered and degraded through autophagy, which helped the cells to survive under stress [15]. As expected, the defective capacity of the old mice liver following I/R was enhanced by the autophagy activator rapamycin. A growing body of evidence has demonstrated the protective role of autophagy, activated by medicines [16], preconditioning [17] and adenoviral gene transfer [18] in ischemic organs. Although it has been established that salubrinal is protective through the inhibition of the ER stress in brain and heart I/R [19,20], to the best of our knowledge, the present results provide the first evidence that salubrinal can protect from hepatic I/R injury through the induction of parkin-dependent mitophagy. In contrast, another study provided the evidence of the detrimental effect of salubrinal in the case of I/R injury [21]. This might be related to the intensity of stress [22] and the crosstalk among autophagy, ER stress and apoptosis [23,24]. Thus, the amelioration of the ER stress may not always be beneficial therapeutically, and caution should be taken in choosing strategies to target the ER stress.

Aging is closely linked to many pathological conditions and functional decline in the heart, brain and liver [11,25]. The age-related decline in the mitochondrial quality control including mitophagy has been demonstrated as a vital early event in organ aging [26]. We uncover that the autophagic protein parkin and Atg5 reduction is a key event culminating in reduced a protective response in an aged liver. Consequently, aged livers exhibit defective autophagosome formation and inefficient or insufficient autophagic flux, impaired mitophagy and mitochondrial failure; these findings are in consistent with those of previous studies [27,28]. In agreement with our findings, parkin has been found to decline in the aorta of old mice and the brain cortex of old monkeys. The expression of parkin is higher in organs with a sufficient blood supply and the susceptibility of cells to parkin deficiency appears to be related to the energy demands. Parkin plays a key role in the ischemia-induced mitophagy process [8,29]. Consist with this, we found that the interfering parkin expression in the L02 cells intensified the cell death and the mitochondrial membrane potential transition onset under the OGD-Rep. condition. The physiological relevance of such a relationship was confirmed and extended *in vivo*, as evidenced by the reversal of the ROS production and the hepatocyte apoptosis through the induction of the parkin expression in the old mice. Similar to our findings, the parkin knockout mice show enhanced susceptibility to myocadiac infarction. In addition, parkin-dependent mitophagy induced by acidic postconditioning rendered the brain resistant to ischemic injury and extended the reperfusion window [30]. Moreover, genipin protected the liver from I/R injury by modulating parkin related mitochondrial quality control [31]. Thus, an enhancement of the parkin expression has a therapeutic potential for ameliorating the age-mediated liver I/R injury.

Kubli reported that the loss of parkin resulted in smaller and more disorganised mitochondria with no adverse effects on the mitochondrial function under the no-stress condition [32]. Our data confirmed that parkin was not critical for the turnover of mitochondria under normal conditions. Parkin-deficient hepatic mitochondria in the old mice’s livers were normal under the base-line conditions but rapidly deteriorated after I/R, suggesting that parkin was involved in maintaining the mitochondrial function in response to stress. Similarly, parkin null mice do not suffer from motor impairments or loss of dopaminergic neurons in the substantia nigra until exposed to stress conditions [33]. Our findings are also consistent with the existing patient data. Loss-of-function mutations in the parkin gene are associated with early-onset familial Parkinson’s disease [26], but there are currently no reports that these patients have an abnormal liver function.

On one hand, the loss of the mitochondrial membrane potential triggers parkin translocation and thereby recruits ubiquitinated proteins to interact with LC3, leading to autophagosome formation around the mitochondrion [34]. Consistent with this, we found that there was an increase in parkin combined with LC3B in the vascular zone of the liver after I/R. In addition, Atg5, a key factor for autophagosome formation, was accumulated in this area. On the other hand, mitochondrial materials such as mitochondrial DNA that leak from damaged mitochondria have been reported to elucidate the inflammatory responses [35]. Our data revealed the infiltration of more inflammatory cells in the mitophagy-defected old mice at 1 h after the reperfusion. The activation of Kupffer cells, regulated by NF-κB, is a central event in the initial phase of hepatic I/R injury [36]. An increased activation of NF-κB was reported in old mice during hepatic I/R [37]. Tran found that NF-κB translocated to the nucleus and bind to the parkin promoter to repress the transcriptional activity in neuronal cells [38]. Additional studies are required to further investigate the role of NF-κB in the mitochondrial clearance in the liver.

Recent studies have provided evidence that ER stress is capable of inducing autophagy in mammalian cells [39,40]. Mitochondria may have the priority to be recognized by an autophagosome under the ER stress evidenced by a recent investigation reporting that autophagosomes form at the contact sites of the mitochondria and the ER [41]. Our data showed that the ER stress response can be activated by I/R, and the phosphorylation of EIF2α was reduced in the old mice. Phosphorylated EIF2α is considered to be a key mediator for the PERK signaling pathway under ER stress. After activation by phosphorylated EIF2α, the downstream transcription factor ATF4 can bind to a specific CREB/ATF site within the parkin promoter [42]. To further reveal such a relationship, we silenced EIF2α and found that the parkin expression was downregulated after OGD-Rep. Interestingly, a recent study documented that transient ischemia triggered a protective ER stress response by upregulating autophagy, while prolonged ischemia induced a pathogenic ER stress response with impaired liver autophagy flux. Moreover, pretreatment with salubrinal inhibited the activation of autophagy and abolished the neuroprotection induced by the ischemic preconditioning of the brain [21]. In our model, salubrinal enhanced the parkin expression and alleviated the hepatic I/R injury by maintaining the phosphorylation of EIF2α *in vivo*. Taken together, the functional dichotomy of stress responses, dictated by the severity of the stress, is dependent on their interactions. The autophagy activity is a key determinant of the outcome of the stress responses in the disease process. The ER stress can augment autophagy at multiple stages [43]. A series of studies have indicated that EIF2α phosphorylation mediates the polyglutamine-induced LC3 conversion via the activation of the Atg5-Atg12-Atg16 complex [44]. However, the linkage between Atg5 and EIF2α was not observed in hepatocytes in the OGD-Rep. induced injury.

Various biochemical and hematological parameters have been identified that, if abnormal within the first few days after the transplant, are associated with relative poor graft outcomes, but their predictive power for an aged graft is poor [45]. Our results provide the first evidence to the best of our knowledge that liver allograft biopsies with parkin expression assessment at 2 h after the reperfusion is associated with the donor age and the peak AST level within 7 days after the transplantation. This reflects that parkin is crucial for identifying these aged patients destined for early graft dysfunction. However, as the sample considered in this analysis was not sufficiently large enough, future studies are warranted for a complete evaluation of the parkin expression as a predictive tool for the aged liver transplantation.

In summary, the present study suggested that the reduced expression of parkin attributed to the increased sensitivity of the old liver to the lethal I/R injury. The phosphorylated EIF2α-regulated parkin expression may be involved in age-dependent mitophagy impairment. These findings implied that enhancing the ER-stress-induced mitophagy could be a novel strategy to improve the liver function of the elderly patients after the liver transplantation.

## MATERIALS AND METHODS

### Animals and establishment of hepatic ischemia-reperfusion injury model

Healthy male C57BL/6 mice at 8-10 weeks (young group) and 12 months (old group) of age, were purchased from the Nanjing University Laboratory Animal Center (Nanjing, China). All the experiments were approved by and conducted in accordance with the ethical guidelines of the Sun Yat-sen University Animal Experimentation Committee and were in compliance with the National Institutes of Health Guide for the Care and Use of Laboratory Animals.

After intraperitoneal injection of 0.6% pentobarbital sodium (100μL/10 g), the 70% liver I/R injury model was performed as previously described by Castellaneta [46]. Ischemia was continued for 60 min and completed by removing the atraumatic vascular clamp. Rapamycin (Sigma 1 mg/kg), chloroquine (Sigma 60 mg/kg) and salubrinal (Sigma 1 mg/kg) were given by an intraperitoneal injection to the mice 1 h before the sham or the ischemia operation. Each treatment group contained 6 mice, which were chosen randomly from the major groups.

### Establishment of OGD-Rep in cell culture

The human hepatocellular line L02 (Guangdong Provincial Key Laboratory of Liver Disease) were cultured in a medium containing high glucose-DMEM with a 10% fetal bovine serum (Gibco). For the OGD treatment, the cells were refreshed with low-glucose (1.5 g/L) DMEM and immediately placed in a HERA cell 150i incubator (Thermos Fisher Scientific Company, UK) at 37°C under hypoxia conditions (5% CO2 and 1% O2) for 24 h. The reperfusion was performed by refreshing cells with normal glucose (4.5 g/L) DMEM and transferred back to the common incubator (21% O2, 5% CO2) for 2 h.

### Additional experimental procedures

For more detailed and additional information on experimental procedures, please see Supplementary Materials and Methods.

### Patient selection and data collection

The data of 46 patients who received the DCD liver transplantation at our centre between April 2016 and January 2017 were collected. The selection criteria were as follows: 1) DCD donor, 2) no severe infection-related or vascular complications, 3) no perioperative period death and 4) no split transplantation. The study protocol was approved by the Clinical Ethics Review Board of the Third Affiliated Hospital of Sun Yat-sen University. Informed consent was obtained according to the Declaration of Helsinki. The biopsy of the allograft was performed 2 h after complete revascularisation.

### Statistical analysis

Data were shown as mean ± SD or presented directly. All the statistical analyses were performed using the Prism statistical software (GraphPad) version 5.0 (USA). A Student’s two-tailed *t*-test was used to compare between the two groups and one-way ANOVA for three or more groups. A probability (*p*) value of <0.05 was considered a statistically significant difference.

## Supplementary Material

Supplementary Materials and Methods

Supplementary Figure
