# Supplementary material for: Aging aggravates hepatic ischemia-reperfusion injury in mice by impairing mitophagy with the involvement of the EIF2α-parkin pathway
Source: Aging (Albany NY). 2018 Aug 8;10(8):1902–20. doi: 10.18632/aging.101511 (PMC6128434; doi:10.18632/aging.101511)
Supplement: Supplementary Materials and Methods [file aging-10-101511-s001.pdf]

SUPPLEMENTARY MATERIAL

SUPPLEMENTARY MATERIALS and METHODS

Histological analysis, TUNEL staining and measurement of mitochondrial superoxide level

The collected livers were fixed with 10% neutral buffered formalin and embedded in paraffin. All the paraffin-embedded sections were stained with H&E for a conventional morphological evaluation. TUNEL staining (Roche Diagnostics, Indianapolis, USA) was used to assess the apoptosis level of the paraffin-embedded fraction slides, according to the manufacturer's instructions. Freshly prepared frozen liver sections were incubated with a 2-µM MitoSOX™ Red mitochondrial superoxide indicator (Invitrogen) for 30 min at 37°C and were observed by fluorescence microscopy and quantified with using the Image pro plus software.

Biochemical analysis

The release of ALT and AST in the serum was measured by standard spectrophotometry using an automated clinical biochemistry analyzer (Olympus AU5400, Tokyo, Japan).

Immunohistochemistry

All the specimens after deparaffinization were rehydrated and a citrate buffer (pH 6.0) was used for antigen retrieval. The hydrogen peroxide solution was used to block the endo-genous peroxidase. The slides after blocking with a 10% normal goat serum were incubated with LC3B (Cell Signaling Technology), TOMM20 (Santa Cruz), parkin (Abcam) and Atg5 (Cell Signaling Technology) at 4°C overnight. After rinsing with PBS, the secondary antibody was added to the slides and incubated at room temperature for 1 h. DAB was added for color development and then counter-stained with hematoxylin. Positive and negative controls were set for each dye batch. For the graft biopsy, the immunohistochemical staining results were assigned the mean score considering both the intensity of the staining and the proportion of the hepatocytes. Each section was independently assessed by two pathologists without any prior knowledge of the patient data.

Transmission electron microscopy (TEM)

TEM was applied to further confirm and monitor autophagy and quantify the autophagic vacuoles. The liver samples were cut into 1 mm^3 and fixed with 2.5% glutaraldehyde in a 0.1 M phosphate buffer at 4℃ (pH 7.4) for 1 h. Ultrathin sections (70-80 nm) were obtained using an ultramicrotome (RMC MT6000-XL) after fixation and dehydration. All the sections were stained with lead citrate and uranylacetate and detected and photographed using a transmission electron microscope (HT7700, Hitachi, Japan).

Immunofluorescence

For the liver tissues, the three processes of dewaxing, dehydration and repairmen were performed on the 4-µm-thick sections. The L02 cells in each group were fixed with 4% paraformaldehyde for 30 min and blocked with 5% BSA for 1 h. Both the liver sections and cells were incubated with the primary antibodies (parkin, LC3B and TOMM20 1:200) at 4℃ overnight. Alexa Flour 488 (1:1000)- and 594 (1:1000)-conjugated secondary antibodies were used for the subsequent incubation for 1 h in the dark at 37℃. Finally, DAPI was used for nucleus staining for 2 min at room temperature in the dark. The samples were observed and photographed using a fluorescence inverted microscope (Leica, Germany). Parkin was purchased from Abcam. TOMM20 from Santa Cruz and LC3B from Cell Signaling Technology. The immunofluorescent second-dary antibodies were purchased from Life, USA.

Cell viability

The measurement of the viable cell mass was performed with CCK8 (Dojindo Laboratories Co., Kumamoto, Japan) according to the manufacturer's instructions. The absorbance of the samples was measured using a microplate reader ELX800 (BIO-TEK Instruments, Inc., Winooski, VT) at 490 nm.

Transfection and RNA silence

The shRNA candidate target sequences to parkin and EIF2a are 5'-CGTGAACATAACTGAGGGCAT-3' and 5'- GAAGATTAACTTTGTGGGAAA-3', respectively. A scrambled shRNA (Scr.) sequence was used as the negative control. The oligonucleotides encoding the parkin-shRNA, EIF2a-shRNA or SCR-shRNA sequence were inserted into the vector (Genechem, China). After successful recombinant virus packaging, the L02 liver cells were infected with the lentivirus carrying the corresponding shRNA. The cells were harvested to assess the efficiency of the gene knockdown by Western blotting.

Mitochondrial membrane potential and measurement of mitochondrial ROS

JC-1 was added to the culture medium (500µL/well) and incubated at 37°C for 30 min in the dark for

mitochondrial staining. After washing twice with a cold dyeing buffer to remove the unbound dye, the samples were resuspended. Quantification by flow cytometry detected mitochondria containing red JC-1 aggregates in the FL2 channel and green JC-1 monomers in the FL1 channel. For the measurement of mitochondrial ROS, the cells were incubated with 250 μL of MitoSox diluted in DMSO (stock solution: 5 mM), for 10 min in darkness at 37°C. The oxidation products were detected using the FL2-H channel of a FACScan flow cytometer.

Western blotting

The primary antibodies included LC3B (Cell Signaling Technology, 1:1000), PARP (Cell Signaling Technology, 1:1000), Caspase 3 (Cell Signaling Technology, 1:1000), TOMM20 (Santa Cruz, 1:1000), ATG3 (total) (Cell Signaling Technology, 1:1000), ATG5 (Cell Signaling Technology, 1:1000), ATG7 (Cell Signaling Technology, 1:1000), Beclin1 (Cell Signaling Technology, 1:1000), PINK1 (Abcam, 1:1000), parkin (Abcam, 1:1000), EIF2α (Cell Signaling Technology, 1:1000), P- EIF2α (Cell Signaling Technology, 1:1000). Thereafter, the secondary antibody (anti-rabbit IgG, 1:5000, Sigma-Aldrich) was used for the 1-h incubation at room temperature. The blots were detected by an enhanced chemiluminescence (ECL) substrate and visualised by FluorChem Systems (Proteinsimple, CA, USA). The intensities of the bands were analysed by a densitometric analysis using an image analyser (Image J software, USA).
