# Supplementary material for: Aging aggravates hepatic ischemia-reperfusion injury in mice by impairing mitophagy with the involvement of the EIF2α-parkin pathway
Source: Aging (Albany NY). 2018 Aug 8;10(8):1902–20. doi: 10.18632/aging.101511 (PMC6128434; doi:10.18632/aging.101511)
Supplement: Supplementary Figure [file aging-10-101511-s002.pdf]

SUPPLEMENTARY FIGURE

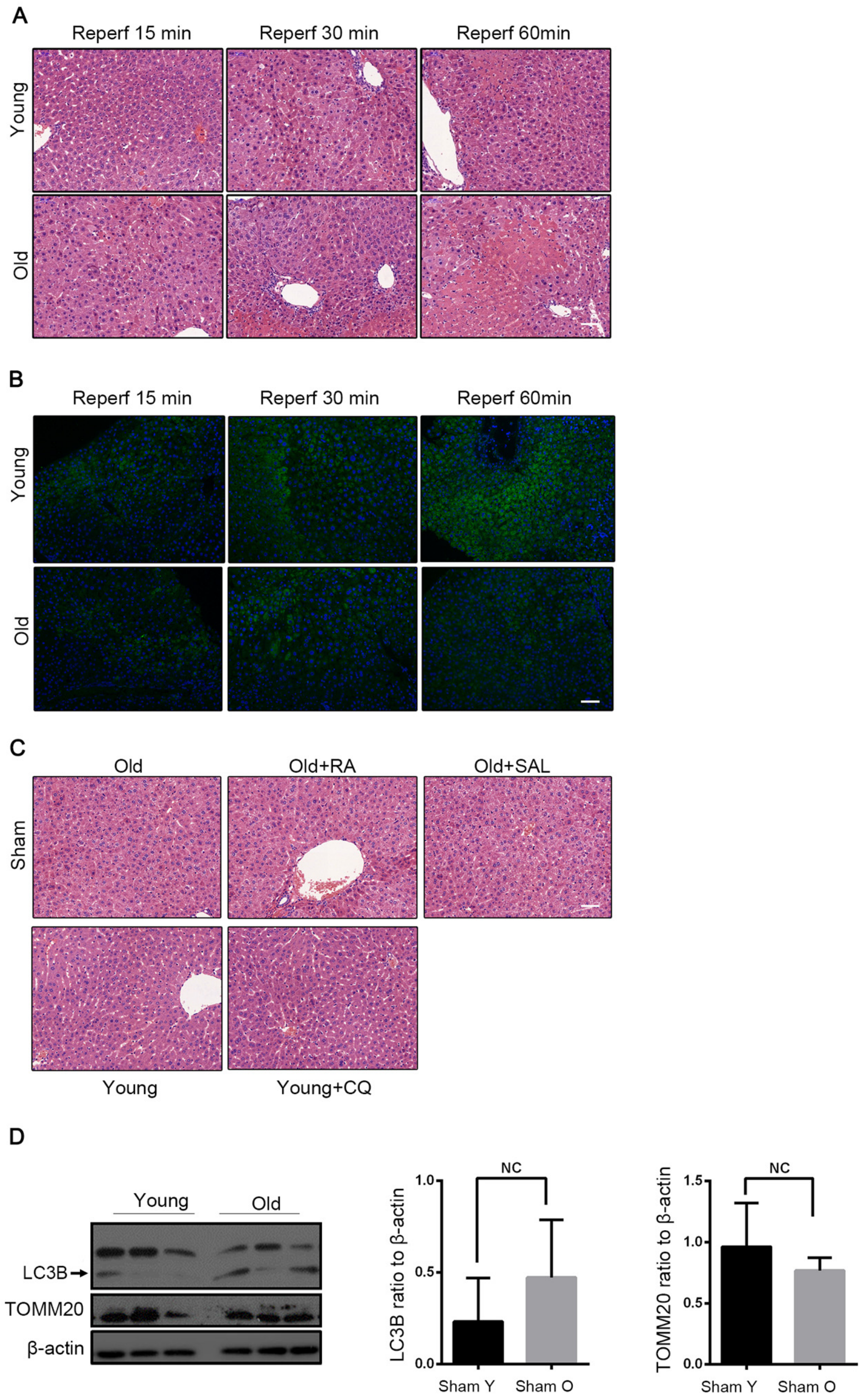

Supplementary Figure 1. Mice of different age were treated as indicated. (**A**) Representative histology of liver by H&E staining at 15, 30 and 60 minutes after reperfusion. (**B**) Representative images of LC3B staining of liver tissues by fluorescence microscopy at 15, 30 and 60 minutes after reperfusion. (**C**) Representative histology of liver by H&E staining from indicated sham group (Y for young mice, Y+CQ for young mice with chloroquine pretreatment, O for old mice, O+RA for old mice with Rapamycin pretreatment, O+SAL for old mice with Salubrinal pretreatment). (**D**) The LC3B and TOMM20 protein levels were determined by western blot analysis from sham group. Scale bar: 50µm.
